# Process evaluation of a stepped-care program to prevent depression in primary care: patients’ and practice nurses’ experiences

**DOI:** 10.1186/s12875-017-0583-7

**Published:** 2017-02-23

**Authors:** Alide D. Pols, Karen Schipper, Debbie Overkamp, Susan E. van Dijk, Judith E. Bosmans, Harm W. J. van Marwijk, Marcel C. Adriaanse, Maurits W. van Tulder

**Affiliations:** 1 Department of Health Sciences and the EMGO Institute for Health and Care Research, Vrije Universiteit, Amsterdam, De Boelelaan 1085, 1081 HV Amsterdam, The Netherlands; 2 Department of General Practice & Elderly Care Medicine and EMGO Institute for Health and Care Research, VU University Medical Centre, Amsterdam, The Netherlands; 3 Department of Medical Humanities, EMGO+ Institute, VU Medical Centre (VUmc), Amsterdam, The Netherlands; 4 CLAHRC Greater Manchester and NIHR School for Primary Care Research, the University of Manchester, Manchester, UK

**Keywords:** Qualitative study, Process evaluation, Major depressive disorder, Subthreshold depression, Stepped care, Diabetes mellitus type 2, Coronary heart disease

## Abstract

**Background:**

Depression is common in patients with diabetes type 2 (DM2) and/or coronary heart disease (CHD), with high personal and societal burden and may even be preventable. Recently, a cluster randomized trial of stepped care to prevent depression among patients with DM2 and/or CHD and subthreshold depression in Dutch primary care (Step-Dep) versus usual care showed no effectiveness. This paper presents its process evaluation, exploring in-depth experiences from a patient and practice nurse perspective to further understand the results.

**Methods:**

A qualitative study was conducted. Using a purposive sampling strategy, data were collected through semi-structured interviews with 24 participants (15 patients and nine practice nurses). All interviews were audiotaped and transcribed verbatim. Atlas.ti 5.7.1 software was used for coding and structuring of themes. A thematic analysis of the data was performed.

**Results:**

The process evaluation showed, even through a negative trial, that Step-Dep was perceived as valuable by both patients and practice nurses; perceived effectiveness on improving depressive symptoms varied greatly, but most felt that it had been beneficial for patients’ well-being. Facilitators were: increased awareness of mental health problems in chronic disease management and improved accessibility and decreased experienced stigma of receiving mental health care. The Patient Health Questionnaire 9 (PHQ-9), used to determine depression severity, functioned as a useful starting point for the conversation on mental health and patients gained more insight into their mental health by regularly filling out the PHQ-9. However, patients and practice nurses did not widely support its use for monitoring depressive symptoms or making treatment decisions. Monitoring mental health was deemed important in chronically ill patients by both patients and practice nurses and was suggested to start at the time of diagnosis of a chronic disease. Appointed barriers were that patients were primarily motivated to participate in scientific research rather than their intrinsic need to improve depressive symptoms. Additionally, various practice nurses preferred offering individually based therapy over pre-determined interventions in a protocolled sequence and somatic practice nurses expressed a lack of competence to recognise and treat mental health problems.

**Conclusion:**

This study demonstrates both the benefits and unique demands of programs such as Step-Dep. The appointed facilitators and barriers could guide the development of future studies aiming to prevent depression in similar patient groups.

## Background

Major depression is estimated to currently affect 350 million people around the world. Depression is the leading cause of disability worldwide and is a major contributor to the overall global burden of disease [1]. People with chronic physical health problems, like type 2 diabetes mellitus (DM2) and coronary heart disease (CHD), are approximately twice as likely to suffer from major depression as compared to the general adult population. Furthermore, when co-occurring, major depression is significantly associated with greater reductions in health status compared with depression alone, or with single or multiple chronic physical conditions alone [2]. It is furthermore increasingly conceptualized as a chronic condition [3].

One approach to reduce the burden of major depression could be to prevent the influx of new cases. Recent meta-analyses have shown that psychological interventions can reduce the incidence of depression, which in high-risk populations can be as high as 25% annually [4, 5]. Offering these in a stepped care format could be an efficient and cost-effective approach to prevent depression, but the evidence is not unequivocal [6]. In stepped care, patients start with minimally intensive evidence-based treatments. Progress is monitored systematically and those patients who do not improve adequately step up to a treatment of higher intensity, thereby making the best use of available resources [7], although the steps perhaps do not make the best use of available clinical expertise [8]. Current evidence on the effectiveness of prevention of depression using stepped care is conflicting. While effective in reducing the incidence of major depressive disorder in some elderly or visually impaired populations [9–11], it was not superior to usual care in other elderly, diabetic or primary care populations [12–15].

We recently performed a randomized controlled trial evaluating a nurse-led stepped-care program to prevent depression among patients with DM2 and/or CHD and subthreshold depression (indicated prevention) in primary care (Step-Dep) in several regions in the Netherlands. Patients with subthreshold depression were identified via screening which is not common practice in Dutch primary care. Our first finding was that both arms had a surprisingly low overall annual incidence of depression (11%). This pragmatic intervention was also not effective in comparison with usual care in our quantitative analyses (Pols AD, Van Dijk, Bosmans SEM, Hoekstra JET, Van Marwijk HWJ, Van Tulder MW, Adriaanse M. Effectiveness of a stepped-care intervention to prevent major depression in patients with type 2 diabetes mellitus and/or coronary heart disease and subthreshold depression: apragmatic cluster randomized controlled trial, submitted). To gain more insight into the facilitators and barriers of the Step-Dep program and to better understand the effects of the intervention in daily life, a qualitative process evaluation study was performed alongside the trial. Qualitative studies can complement the quantitative outcomes by gaining deeper understanding of interventions and this can yield valuable input for the development and implementation of the next generation of care models in the management of mental-physical multimorbidity and frailty, and can inform the policy debate [16]. This paper reports the results of this process evaluation exploring experiences with the Step-Dep program from a patient and practice nurse perspective.

## Methods

### Step-Dep study

The process evaluation entailed semi-structured face-to-face interviews with both patients and practice nurses in the intervention arm of the Step-Dep study. The methods and design have been described previously [17]. In short, we screened all patients with DM2 and/or CHD in 27 participating General Practitioner (GP) practices for subthreshold depression, defined as a Patient Health Questionnaire 9 (PHQ-9; range 0–27) score of six or more [18, 19], and no major depressive disorder according to the Mini International Neuropsychiatric Interview (MINI) [20, 21]. Patients in the intervention arm were offered a stepped care preventive program, and patients in the control arm received care as usual. The stepped care intervention consisted of four sequential but flexible treatment steps, each lasting 3 months; 1) watchful waiting, 2) guided self-help, 3) problem solving treatment (PST) and 4) referral to the general practitioner. After each step, patients with a persisting PHQ-9 score of six or more were offered the next treatment step of the intervention. Due to the pragmatic nature of the Step-Dep trial, treatment steps could be personalized or skipped if deemed necessary. A trained practice nurse delivered the stepped care program. This training focused on how to implement the stepped-care program, how to provide guidance with the self-help course using motivational interviewing techniques and how to provide the PST (see Appendix 1 for a detailed description of patient an practice nurse roles during Step-Dep). The training was developed and provided by a qualified trainer in collaboration with research team members. During the trial, all practice nurses were regularly supervised by the training staff and could contact them to discuss any questions or problems.

### Participant selection and recruitment

We used purposeful sampling in order to include patients with as many perspectives on the pre-specified topics as possible [22]. Based on a literature review of factors influencing depression incidence and outcome, we selected patients on: gender, age, presence of DM2 and/or CHD, self-reported history of depression, self-reported current depression, level of education, baseline depression severity (PHQ-9), baseline anxiety severity (HADS-a), baseline quality of life score (EQ5D), baseline social support scores, and locus of control scores. We also selected patients who had received different elements of the stepped care program as well as a patient that had dropped-out of the program. We included patients from all different GP practices.

All nine practice nurses involved in the actual implementation of the Step-Dep program were interviewed. Amongst them were both somatic practice nurses, whose primary task is the physical health management of primary care patients with diabetes and/or cardiovascular disease, and psychological practice nurses, whose primary task is to provide low-intensity mental health care for primary care patients. In the Netherlands, the educational programs for these two types of practice nurses are separate and generally take 1 year after an appropriate pre-registration education of 4 years at an University of Applied Sciences. Patients are not charged for practice nurse consultations in primary care; this type of care is reimbursed within public health insurance. At the start of the intervention in 2013, per standard practice size of 2350 registered patients, 0.33FTE somatic practice nurse and 0.25FTE psychological practice nurse were available.

Patients and practice nurses were asked by an investigator (ADP or DO) to participate in the evaluation of the Step-Dep study by phone. All selected participants agreed to be interviewed, except for three patients due to terminal illness of themselves or their partners.

### Data collection

To structure the interviews and maintain conformity in the different interviews, a topic guide was used (Appendix 2), which was developed based on study aims and patients’ and practice nurses’ feedback during the Step-Dep study. Additionally, to systematically evaluate the experiences with the Step-Dep program, we added questions based on the RE-AIM model [23]. RE-AIM assesses five dimensions of an intervention: reach, efficacy, adoption, implementation, and maintenance. Reach explores characteristics of study participants compared to the target population; efficacy refers to whether the targeted outcome was achieved; adoption assesses variables of the staff and settings executing the intervention; implementation refers to intervention fidelity and resources (i.e. time); maintenance evaluates both individual-level and organizational/setting-level intervention sustainability. Based on the topic guide, semi-structured open-ended questions were formulated (Appendix 3 and 4). The process of data collection and analysis was iterative, meaning that the researchers started data analysis after the first interviews to further explore and validate emerging themes in the next interviews. This process evaluation was summative and retrospective; the results were not used to adjust the program along the way.

All interviews were conducted between September and November 2015 by ADP and DO. Interviews with individual participants were held at home (patients *n* = 11), at the GP practice (practice nurses *n* = 8) or at the Vrije Universiteit Amsterdam (patients *n* = 4, practice nurses *n* = 1). The interviews lasted about 45 min each. The interviews were audio-recorded and transcribed verbatim with the permission of the participants. To check the validity of the transcription, participants received a summary of their interview and were asked if they recognized the main themes (member check) [22]. All participants but one (a patient who could not be reached despite multiple attempts) confirmed the content of the summary by mail to be representative for the interview.

### Data analysis

The transcriptions of the interviews were analysed by two researchers (AP and DO) and emerging themes and subthemes were identified individually. First, using Atlas.ti 5.7.1 software codes and labels were attached to citations related to specific topics (open coding), leading to a set of descriptive topics per transcript. Then, all labels of all transcripts were compared and redefined, and clustered into themes and subthemes (axial coding). Eventually, overarching themes were formulated (selective coding), and similarities and differences between cases were identified (cross case analysis of constant comparison) to provide further insight into the research questions. Furthermore, ‘check coding’ was used, meaning that three different researchers (AP, DO and KS) were involved in the process of data analysis, in order to enhance the reliability [22]. Relevant themes were agreed upon and for each theme the most illustrating quotes were selected and only adjusted if necessary for readability for the final report.

### Data saturation

In qualitative research, the process of data collection and analysis ends when ‘saturation’ is reached [22]. This is the point where no new information is added and data replication occurs. From the patient perspective, we reached this point after interviewing 11 patients. We have subsequently interviewed four more patients to confirm this.

## Results

### Description of participants

Of the participating patients, eight were female and seven male. The age range was from 48 to 84 years. PHQ-9 levels at baseline varied from 2 to 16. Additional data regarding the chronic condition, education level, self-reported depression at baseline, self-reported history of depression, and number of program steps terminated can be found in Table 1. Of the interviewed practice nurses, six were psychological practice nurses and three were somatic practice nurses. One of the three somatic practice nurses had been a psychological practice nurse before. The number of Step-Dep patients per practice nurse varied from 3 to 24. To ensure anonymity, age and gender are not mentioned.

**Table 1:** Participant characteristics

Patients									
Interview nr	Age	Sex	DM2/CHD	Educational level	Self-reported depression at baseline	Self-reported history of depression	PHQ-9 score at inclusion	PHQ-9 score at baseline^a^	Number of program steps terminated
P1	66	f	CHD	high	no	yes	7	5	2
P2	61	f	CHD	high	no	yes	7	4	1
P3	63	f	Both	intermediate	yes	yes	9	16	referred
P4	84	f	CHD	low	yes	no	10	11	3
P5	53	f	DM2	high	no	yes	16	14	2
P6	72	m	CHD	intermediate	no	yes	10	2	1
P7	56	m	DM2	high	no	yes	10	10	3
P8	73	f	Both	low	no	no	11	6	1
P9	55	m	Both	intermediate	no	yes	14	12	4
P10	48	m	DM2	intermediate	yes	yes	12	12	1
P11	61	m	DM2	low	yes	yes	8	4	Drop-out
P12	56	f	Both	high	yes	yes	14	12	3
P13	66	m	CHD	high	no	yes	7	5	referred
P14	57	m	DM2	intermediate	no	no	14	7	3
P15	55	f	CHD	low	no	no	15	8	4
Practice nurses									
Interview nr			Practice nurse type				Number of Step-Dep patients treated		
N1			Psychological practice nurse				24		
N2			Psychological practice nurse				15		
N3			Psychological practice nurse				13		
N4			Psychological practice nurse				10		
N5			Somatic practice nurse				3		
N6			Somatic practice nurse				6		
N7			Psychological practice nurse				15		
N8			Currently somatic practice nurse, previously psychological practice nurse				3		
N9			Psychological practice nurse				7		

*Abbreviations: F* female, *M* male, *CHD* Coronary Heart Disease, *DM2* Diabetes Mellitus type 2, *PHQ-9* Patients Health Questionnaire 9 score

^a^Scores do not equal inclusion PHQ-9 scores due to time between inclusion and baseline

### Themes

The results from this study can be understood using five overarching themes; 1) motivation to participate, 2) the Step-Dep program, 3) patient care, 4) patient wellbeing and 5) recommendations for future care. They illuminate the main experienced facilitators and barriers of Step-Dep. The term facilitator used in this paper translates to what interviewees named as experienced successful, useful, effective or strong elements of the program. The term barriers is used to express the opposite. An overview of the main results per theme can be found in Table 2.

**Table 2:** Overview of main results by theme

Motivation to participate
• Patients were primarily motivated to participate in Step-Dep to contribute to scientific research rather than having a desire to improve their depressive symptoms
• Practice nurses perceived this as a barrier to motivate patients for the different treatment steps, especially the self-help course
The Step-Dep program
*Role and competences of the practice nurse:*
• In order to discuss their mental health problems, patients needed to feel a connection to the practice nurse
• Somatic practice nurses expressed a lack of competence to recognise and treat mental health problems
*Treatment steps and stepped care protocol:*
• The offered treatments were viewed to be only suitable for specific patients
• Practice nurses preferred flexibility in the choice of therapy over pre-determined interventions in a one-size fits all protocol
*Using the PHQ-9:*
• The PHQ functioned as a useful starting point for the conversation on mental health, but was not widely supported as monitoring instrument or to base treatment decisions on
Patient care
• Interviewees experienced improved accessibility and decreased experienced stigma of receiving mental health care
• The increased awareness and attention for mental aspects in chronic disease management were experienced as very valuable
• Monitoring mental health is deemed important
Patient wellbeing
• Patients gained more insight into their mental health status by regularly filling out the PHQ-9
Recommendations for future care
• Monitoring of mental health in chronically ill patients should start from the time of diagnosis of the chronic disease

#### Theme: motivation to participate

Patients reported widely different reasons to participate. Interestingly, less than half named the desire to improve their mood as a primary motivation. A few wanted to use the study to analyse their mood, whereas others were curious about the possible interaction of their depressive symptoms with their chronic disease or felt that the study acknowledged this link. Another reason mentioned, was the GP’s advice to enrol.

All interviewed patients stressed the importance of contributing to scientific research, and named this as (one of) their main motivator(s) to participate.

*“In my opinion, if you can get certain results from a research program like this, that could help other people, you should collaborate.” (P14)*

Practice nurses picked up on this issue when treating Step-Dep participants and perceived it as a barrier to motivate some patients for the different treatment steps. Especially with the self-help module, a treatment which requires a relatively large input from patients, this lack of intrinsic motivation was perceived as problematic.

*“By many, this wasn’t actively requested. They were asked: ‘Would you like to participate in a research program?’ It did not come from within, like: ‘I am stumbling upon problems, I am stuck, I want help.’ It’s a different story if it would originate from intrinsic motivation.” (N1)*

This phenomenon might explain some of the lack of effectiveness and the relatively low uptake of the Step-Dep intervention. Around 30% of patients who were offered one of the treatment steps, declined this step (Pols AD, Van Dijk, Bosmans SEM, Hoekstra JET, Van Marwijk HWJ, Van Tulder MW, Adriaanse M. Effectiveness of a stepped-care intervention to prevent major depression in patients with type 2 diabetes mellitus and/or coronary heart disease and subthreshold depression: a pragmatic cluster randomized controlled trial, submitted). Possibly, the research setting created an artificial situation, motivating patients to participate without much need for care.

#### Theme: the step-Dep program

##### The role and competences of the practice nurse

The patient interviews illuminated that a good personal connection with the practice nurse determined whether they felt they could discuss their mental health problems. For several patients, the contact with the practice nurse itself even was the most important facilitator of the program, whereas for two others the lack of a good connection was the most important barrier.

*“The best element…that (name practice nurse) listened to me so carefully […] He made me feel calm […] we just got along well.” (P4)*

*“If I am with someone, with whom I can easily talk and I feel like he understands me and we click, I can open up more. I did not feel like I was really able to do that now.” (P10)*

Reflecting on their role and competences as mental health caregivers, the somatic practice nurses who lacked mental health work experience, cited that they lacked education, skills and experience to recognise and treat mental health problems in general, despite the Step-Dep training. They pointed this out as the main barrier to participate and function in the Step-Dep program.

*“That (mental health care for chronic illnesses) is not something you learn during the practice nurse educational program. […] Purely somatic health.” (N5)*

*“As a somatic practice nurse, I felt like I utterly failed these people in certain aspects. They clearly indicated that they were dealing with problems and that they were struggling […] I felt like I wasn’t really able to help out.” (N6)*

They did want to master these competences, since in their experience, various mental health problems often interfere with somatic problems and they found these skills essential for a holistic treatment.

*“I have often noticed that the insulin dependent patients are very afraid of injections or hypoglycaemia. There is a lot of anxiety involved. There are lots of people who just don’t want to exercise. That is partly lifestyle, but also often psychological. People who are severely overweight are better off being referred to a psychologist than to a dietician. I feel that one of the most imperative things to do then is to offer psychological help, but as a somatic practice nurse, you need more education on how to do that and how to recognise such cases.” (N5)*

All practice nurses felt that the PST training was very useful and informative. Even so, the delivery of the PST was frequently experienced as difficult. Two somatic practice nurses referred their patients to the psychological practice nurse for this step *“Because I did not feel competent, I reckoned it required more know-how.” (N5)*

Even psychological practice nurses said that in order to work with PST with every possible patient, they would require more practice. Since they only had a few patients that required PST within Step-Dep, these skills could not be extensively trained.

*“I had that training and found it very useful; I learned a lot from it. Before I would be able to fully use it in daily practice at work, I would like some extra training.” (N2)*

##### The treatment steps and protocol

Practice nurses found it easy to work with the simple and straightforward Step-Dep protocol and deviations were hardly deemed necessary. However, ideally, they would prefer more room for their own choice of therapy, based on the estimated needs of a patient, instead of pre-determined steps in a protocolled sequence. Some practice nurses did not find the watchful waiting step fitting for patients they considered in need of care and would have preferred to skip this step. Also the self-help and PST were viewed as suitable for specific patients only.

*“I would like to have a little more freedom of choice though.” (N3)*

*“You should always keep in mind if it matches one’s individual level and learning style and consider carefully if it will be of use to someone.“ (N9)*

In line with the findings from the practice nurses’ interviews, the experienced usefulness for patients was mixed for both the self-help and PST. Half of the interviewed patients that were offered the self-help course considered it one of the best elements of the Step-Dep program, whereas the other half did not find it helpful. Commonly mentioned benefits were the practical advices it offers, the accessible and understandable writing style, the insight it provided into their symptoms, and the advantage of being able to look up information at their own convenience later on. Barriers were how the course confronted them with their negative current mental state and the seemingly overwhelming amount of information.

*“It is difficult to face at times. […] To admit that you have a problem and then pick up that book.” (P9)*

Patients figured that more intensive guidance from the practice nurse was helpful to comply with the course *“…in order to be forced to actually go through that book…” (P9)* and to reduce the amount of information by recommending specific chapters applicable to them.

*“(Name practice nurse) would say: ‘Let’s go to chapter 9; it is precisely what you have been through. Why don’t you read that so we can go from there.’ That helps.” (P7)*

Most practice nurses also experienced that their guidance, especially in combination with using motivational interviewing techniques, did improve patients’ motivation, which could be a barrier as mentioned in the previous theme.

Three of the interviewed patients had received PST and their experiences were again quite personal. One patient felt it was too similar to his work approach and this therapy therefore did not work for him.

*“[…] that is how you approach a project. It gets on my nerves if you analyse your own health in that same, simplistic manner.” (P7)*

The other two patients felt it did help to deal with their problems, although it was sometimes hard to face them.

*“To look at it differently, from another perspective. It made me face my problems. It did help me, but not all the time, because I can’t just change like that.” (P12)*

### The PHQ-9 questionnaire

During the Step-Dep study, the PHQ-9 questionnaire was used for screening and monitoring of depressive symptoms and treatment decisions were based on its scores. A positive side-effect was that the majority of both patients and practice nurses felt that filling out the PHQ-9 together was an easy starting point to talk about patients’ mental state.

*“Filling out the questionnaire is convenient, because it is a natural way to start conversation.” (P5)*

Two patients, however, felt that their 3-monthly sessions were limited to this purpose, which was dissatisfying for them.

When investigating patients’ experiences with how accurately their scores on the PHQ-9 questionnaire reflected their depressive symptom severity, all but two interviewed patients recognized themselves in the (subthreshold) depressed profile at the start of Step-Dep. However, four patients felt that the scores during the 1-year follow-up did not measure the change in their mood accurately.

*”When she would say: ‘Your score has improved since last time’, I would feel like: ‘Ok, if you say so.’ It did not feel like it had improved.” (P14)*

In concordance with these patients, most of the practice nurses did not feel that the PHQ-9 measured changes in depressive symptoms during the year of follow-up accurately and making treatment decisions solely based on the PHQ-9 was not considered sufficient; clinical judgement was deemed necessary.

*“You should always look at the PHQ as a whole. […] I don’t think you should ever use an instrument like that to make a stand-alone decision or base that on a certain score.” (N3)*

### Theme: patient care

Due to the preventive approach, meaning that care was pro-actively offered as standard care, many patients and practice nurses experienced improved accessibility of mental health care. Especially the advantage of receiving care without a stigma was expressed.

*“I had been depressed, but I was doing a bit better. I thought: ‘If I participate and get this help, it won’t be so hard to deal with.’ You won’t say: ‘Ring the alarm, I need help because I am not well mentally.’” (P7)*

*“A low threshold. An easy access without a stigma. […] Like this, it is offered as ‘This is standard care for this group.’ This way you are not crazy, it is not all in your head, it is just standard care.” (N4)*

Many patients said that during the care for their chronic condition, no or very little attention is ever paid to mental health.

*“I have had that chronic disease for a long time now. No attention was ever paid to it, mentally. It was just like: ‘Well, you have diabetes. You can’t do this, but you can do that. Take some pills and that’s it.’” (P14)*

Participation in the Step-Dep study also made somatic practice nurses realise that normally, they put all emphasis on physical and none on mental health.

*“What I liked, was that every 3 months you would ask: ‘How are you? How are you feeling mentally?’ That actually never comes up during diabetes treatment.” (N8)*

The psychological practice nurses considered this increased awareness and attention on mental health an important improvement of current chronic disease management.

*“Just paying that explicit attention; ‘How did things evolve for you, ever since you knew you had diabetes or heart failure?’ That was received positively by many, and had been missing as well. Many people had to cope with it on their own, where an intervention might have been necessary. A project like this makes us health care providers more aware of the fact that we should monitor closely what the effect of our bad news will be.” (N1)*

One other highly valued aspect of the Step-Dep intervention was that the practice nurse visits were every 3 months. Both patients and practice nurses felt that this monitoring of depressive symptoms was beneficial; it felt like a safety net.

*“What worked for me was that because of the consultations with (name practice nurse), someone was keeping an eye on me.” (P5)*

*“Especially those who weren’t doing all too well and who were struggling a bit, which doesn’t necessarily lead to a depression, but is quite difficult, those were pleased to be monitored so closely.” (N3)*

However, a few patients felt it was an extra burden to come to the GP practice every 3 months during a year, but at the same time said it was necessary to adequately monitor their mental health state.

*“It is indeed strenuous and a year seems like a lot at the beginning, but I do think you actually need that.” (P7)*

Part of this burden might have been caused by the research setting, since patients also had to fill out 3-monthly online questionnaires containing the HADS, PHQ-9, EQ5D, TIC-P, locus of control and perceived recovery questionnaires.

### Theme: patient wellbeing

Remarkably, almost all patients indicated that they gained more insight into their mental state just by regularly filling out the PHQ, which they pointed out as one of the most important benefits of the program. Taking the time to analyse their mental state and depressive symptoms served as a form of self-reflection.

*“The real eye-opener for me was that I became aware of my own behaviour. As I filled out the questionnaires again and again, I realised: ‘This is how people see me.’ Whereas I hadn’t really noticed my negativity or that I was feeling a bit down myself. That was the biggest plus for me.” (P10)*

The majority of interviewed patients and practice nurses felt that Step-Dep had been beneficial for patients’ wellbeing somehow, however sometimes in other ways than improving depressive symptoms per se.

*“Just talking about depression or stress, that by itself is so useful. Not to make it go away, but to keep it under control, to be heard or to feel supported.” (P1)*

*“They mainly benefitted from being more aware of and having more insight into how a chronic disease like diabetes or heart-failure can affect how we function and feel. And the acknowledgment; somebody is really taking it seriously and listening carefully.” (N1)*

Almost all patients would therefore recommend others to participate in a program like Step.

*“With very little investment, it might become clearer to you what your problem is and be of help.” (P11)*

When evaluating the perceived effectiveness on improving depressive symptoms of the program, it became apparent that this varied greatly between patients. Several felt that their depressive symptoms had evidently improved because of Step-Dep, whereas a few said that the program had made no difference at all.

Practice nurses viewed this in a similar way, saying that *“… some really benefitted from it and others did not.” (N4)*

Almost all practice nurses had several examples of patients where they felt that Step-Dep had really improved depressive symptoms.

*“I definitely see positive results. I see, and that is also how they describe it, that they have more tools, a big repertoire of possible solutions to try out when they are not doing well. They have more control over their problems.” (N9)*

When investigating why for some the program had not been useful, explanations were diverse. For some patients, the fact that they were participating in a scientific research lowered the expectations of possible benefits from the program, which seems to have formed a barrier for effective care for these patients.

*“Because the program is called ‘research’, you don’t expect it to offer help. […] I’ve just always looked at it as research.” (P13)*

Others did not get practical advices on how to improve their mood, the treatment they wanted or enough treatment sessions. One patient disengaged from the program because it did not help him, whereas a work reintegration project he was simultaneously engaged in did, because of the purpose of and link to coming back to work.

When questioning why the practice nurses felt that the program had not been useful for some patients, explanations were just as diverse. One said the program had been offered too late. Another said that it was hard to really measure the experienced benefits.

*“I think that most people benefitted from it in some way, but that is very so hard to measure.” (N2)*

Two practice nurses concluded that the program sometimes did not work because many patients said they did not have any depressive symptoms.

*“Almost everybody said that they had no depressive symptoms. So you can ask yourself whether you’ve reached the target population you were aiming for.” (N2)*

A program like Step-Dep was felt not to be suitable for patients with psychiatric comorbidity, which was indeed an exclusion criterion. Nonetheless, two practice nurses did have such patients in their Step-Dep treatment and reckoned that to be the reason why these patients did not improve during the program.

*“If people have a lot of mental baggage, this doesn’t work sufficiently. […] If you would exclude these vulnerable individuals, I think it would work very well.” (N4)*

### Theme: recommendations for future care

When discussing future prevention of depression, both patients and practice nurses agreed that this should take place in the GP practice. Patients prefer having chronic disease management clustered in one facility and enjoy the familiarity with the caregivers present in a primary care practice. Both a patient and a practice nurse suggested offering a prevention program like Step-Dep at the time of a new diagnosis of a chronic disease.

*“Caring for people who are confronted with this for the first time, by their general practitioner, could be a good idea.” (P2)*

*“We were too late for a very large part of the target population. Because some had already been through a phase of excessive sadness, which made me think: ‘We might have been able to avoid this by offering a program like Step-Dep earlier on.’” (N1)*

When testing this idea with others patients and practice nurses, most viewed it as an improvement of current chronic care. A few practice nurses added that one should avoid unnecessary medicalization of patients. In their opinion, patients should be made aware of the possibilities of mental health care, but only start therapy when experiencing problems.

## Discussion

This qualitative study reports the results of a process evaluation exploring experiences with the Step-Dep program from a patient and practice nurse perspective. Our findings show that the main facilitators were: increased awareness of and insight into mental health in chronic disease management and improved accessibility and decreased experienced stigma of receiving mental health care. Main barriers were that patients were primarily motivated to participate to contribute to scientific research rather than their intrinsic need to improve depressive symptoms. Additionally, the PHQ-9 was not widely supported to monitor depressive symptoms or base treatment decisions on. Furthermore, somatic practice nurses expressed a lack of competence to recognise and treat mental health problems.

This process analysis has several strengths. Interviewing both patients and practice nurses enabled an evaluation from two essential perspectives; caregiver and care receiver. These perspectives were assessed within the context of a pragmatic trial, approximating a routine setting as much as possible. Additionally, the use of the theoretical RE-AIM framework in this evaluation ensured a thorough investigation of barriers and facilitators. Other strengths are the utilisation of two independent analysts, the systematic development of codes and code definitions, the use of a qualitative computer program, and complete data saturation of information after coding the interviews.

Limitations of this study are that one of the researchers conducting the interviews was also one of the main researchers of the Step-Dep program, possibly influencing interviewees’ answers. However, before starting the interviews, strong emphasis was put on the importance of all feedback to improve future depression care. Our findings may not generalise to contexts where chronic disease management relies on different professionals than the GP and the somatic practice nurse, or where psychological practice nurses are not co-located or available in primary care. Furthermore, due to the aim of this process evaluation, we only interviewed patients from the intervention arm. Information from the control arm patients, on for example their own ‘self-help’ strategies and experiences with filling out the PHQ-9, could have been of added value. Considering the strengths and limitations of this study, our findings give important input for future research. We will discuss the principal findings in the light of current literature.

Firstly, the improved accessibility of care and the perception of a less stigmatising way in which care was delivered are in line with findings from another qualitative interview study among patients, GPs and practice nurses performed alongside a comparable randomised clinical trial on depression care [24]. Such benefits are important to patients, but hard to measure and usually not evaluated in effectiveness studies.

Secondly, it seemed of added value to standardly use the PHQ-9 in chronic disease management to offer patients a form of self-reflection on their mental state and facilitate the conversation on this topic. In another study, patients also saw it as an efficient and structured supplement to medical judgment, and as evidence that general practitioners were taking their problems seriously [25]. Even though the PHQ-9 has been shown to be a valid instrument to both screen for [18, 19] and monitor depressive symptoms [26], many patients and practice nurses in our study did not find this instrument appropriate for the latter. The preference of caregivers to rely on clinical judgment rather than depression severity scales has been described before [25]. Possibly, the most acceptable use of the PHQ-9 for caregivers would be as an instrument of self-reflection and as an ‘ice-breaker’ , but not to base treatment decisions on.

Thirdly, our data revealed that somatic practice nurses experience a lack of competence in recognizing and handling depressive symptoms in chronically ill patients. Other qualitative studies have observed the same [24, 27, 28]. While for both somatic and psychological practice nurses the competence with the delivery of PST seemed dependent on the number of patients treated in Step-Dep, this general lack of confidence for the somatic practice nurses did not. The 2-day Step-Dep training appeared insufficient to compensate for experienced educational shortcomings. Despite the small number of somatic practice nurses in this study, this is an important finding given the prevalence of depressive symptoms in chronically ill patients and the increasing lead of the somatic practice nurse in chronic disease management. Therefore, we consider it important to educate somatic practice nurses better in recognizing and handling mental health problems. The interviewed somatic practice nurses in this study felt this need and were willing to do so. In contrast to our findings, the study by Barley et al. [29] showed that practice nurses do not see mental healthcare as part of what they do. In addition, a recent qualitative study on integrated care from the UK indicated that patients might prefer not to discuss mental health problems with their somatic health caregiver [24]. However, this was not in line with our study outcomes, where this preference was not expressed by patients. To explain these differences and to determine potential improvements in the somatic practice nurse education on mental health and how to best offer future integrated care, these views and preferences should be evaluated further.

Finally, most interviewed patients said to have been motivated to participate in order to contribute to scientific research, which practice nurses perceived as a barrier to deliver optimal care. It is possible that patients were not sufficiently aware of the possible benefits of the intervention for their depressive symptoms. However, both this potential benefit and the positive screening result on subthreshold depression were explicitly mentioned in two separate letters and in the final phone call during the consenting process, and during the first practice nurse visit both the rationale of Step-Dep and the depressive symptoms were discussed as well. It seems more plausible that the extent to which patients experienced a need for the offered mental health care played a role. Having a need for care is an essential motivator to take up offered care, especially in view of the self-activating nature of the offered psychological interventions within the Step-Dep study. Since prevention is often offered to people in an early or mild stage of their mood disorder, implicating less distress or suffering of the patient and therefore a limited need for care [30], this barrier may be challenging to overcome. The depression guidelines of the Dutch College of General Practitioners stress the importance of the existence of the need for care to increase the probability of treatment success, and plead against pro-actively offering depression care to patients having depressive symptoms as indicated by screening [31].

Further research should focus on approaches within chronic disease management to identify and proactively treat only those who are likely to benefit from preventive depression care but also experience a need for such care. Finding optimal strategies to routinely assess and monitor mental health issues while supporting resilience is perhaps required for the rest.

## Conclusion

Although Step-Dep was not superior to care as usual in the prevention of major depression, it was perceived as valuable by the interviewed patients and practice nurses. The perceived effectiveness on improving depressive symptoms varied greatly among interviewees, but most felt that the program had been beneficial for patients’ well-being. Main facilitators, such as increased awareness and understanding of mental health problems, improved accessibility and decreased experienced stigma of mental health care in chronic disease management are difficult to capture in conventional quantitative outcomes. These difficulties in combination with the appointed barriers may have contributed to the non-significant difference in effects of the Step-Dep intervention compared to usual care. Notwithstanding, both the facilitators and barriers described in this process evaluation might guide the development of future studies aiming to reduce the burden of depression among patients with a chronic physical disorder.

## Appendix 1

**Table 3:** Practice nurse and patient roles per step in the Step-Dep program

Step	Role of practice nurse	Role of patient
1. Watchful waiting	Introductory consultation with patient. Explains stepped-care program and its rationale. If applicable, gives information and/or brochure about mild depression with simple advices on how to cope with mild depressive symptoms. Is available for patient, if needed	Gets acquainted with practice nurse. Receives information on stepped-care program and its rationale. Contacts practice nurse if needed
2. Guided self-help	Explains self-help course, hands out materials. Contacts patient every other week by phone to monitor progress. Uses motivational interviewing techniques to activate the patient, if needed. If needed, invites patient to discuss current depressive symptoms; if needed offers early progress to step 3	Starts self-help and works through course at own convenience. Discusses progress every other week. If needed, visits practice nurse and starts step 3
3. Problem Solving Treatment	Offers brief cognitive behavioral intervention focusing on practical skill building in 7 sessions. Explains stages of problem solving and applies to problems encountered in daily life, helping to regain control of life	Visits practice nurse for 7 PST sessions, working through problems together, learning practical skill building
4. Referral to GP	Refers patients to GP for further assessment of depressive symptoms. Provides a summary of the offered treatment	Visits GP to discuss provided treatment and following treatment for depressive symptoms

## Appendix 2

**Table 4:** Topic list

RE-AIM	Topic
Reach	Appropriateness Step-Dep patients (target population)
	Depression: recognition, severity, causes, improving factors
	Need for care
	Motivation to participate
	Access mental health care
Efficacy	Perceived effectiveness
	Perceived usefulness
Adoption	Information practices, caregivers
Implementation	Barriers & facilitators
	Deviations from protocol
	Reasons for dropout
	Prerequisites for implementation
Maintenance	Satisfaction
	Feasibility for future

## Appendix 3

**Table 5:** Patients interview

Topic	Question
General	How was your experience participating in Step-Dep/the program in your general practitioner practice?
	What was the best part for you?
	What was the weakest part for you?
Motivation	Why did you decide to participate in Step-Dep?
Mental state	How would you describe your mental state before starting Step-Dep? If not depressed: please tell more about it?
	If depressed: please tell more about it? Did it influence your life? What do you think caused it? Is there a relationship with your chronic disease? How? How is your mental state now? If improved: what are the reasons for that improvement?
	Did you feel the PHQ-9 reflected your mental state correctly? Why? Why not?
Need for care	Were you in need of care/a preventive program to improve depressive symptoms?
	How would it have been, if you had not received an invitation for Step-Dep?
	What were your expectations/hopes from the program?
	Did the program match your needs?
	What would your care of choice have been like? And to improve depressive symptoms?
	How would it have been for you to be offered a program at the time of diagnosis of your chronic disease?
Perceived effectiveness	Was the offered program useful to improve your depressive symptoms? Why? Why not? What was most useful to you? How do you see that in the long-term?
	How were/was the consultations with the practice nurse/self-help/problem solving treatment/referral to general practitioner for you?
Suggestions for future care	Would you recommend this program to others? Why? Why not? To whom?
	What would your suggestions be to improve Step-Dep?
	Is there anything you would like to add to the interview?

## Appendix 4

**Table 6:** Practice nurse interview

Topic	Question
General	How did you experience executing Step-Dep?
	What is your opinion on the Step-Dep program?
	What were the main facilitators?
	What were the main barriers?
Reach	Were the selected patients appropriate for this prevention program? Why? Why not?
	How did you view their mental state/depressive symptoms? Did patients recognize themselves in the depressed profile? What are causes for depressive symptoms? How do you view the relationship with the chronic disease? What coping strategies do patients have with a chronic disease?
	Were the patients in need for care for depression? Other need for care? Why? Why not?
Efficacy	Did the program match their need for care?
	Was Step-Dep effective in your opinion on preventing depression/improving depressive symptoms for these patients? Why? Why not? How?
	What is your view on the program elements: consultations, self-help, problem solving treatment, referral to general practitioner?
	If the depressive symptoms improved in your patients; what was the reason for this improvement? Did the program play a part?
Implementation	Why did you decide to participate in Step-Dep?
	How do you view your competences to execute the program?
	Was it necessary to deviate from the protocol? Why? Why not?
	How was using the PHQ-9 for you? And as a screening/monitoring/decision tool?
	How much time would you need for the consultations/self-help/problem solving treatment?
Maintenance	Is this program (or elements) useful in daily practice for this group? Why? Why not?
	Would you use this program (or elements) in the future? Why? Why not?
	What would be necessary to implement this in your practice?
	How would you ideally see depression prevention?
	What is your opinion on offering a program like that at the time of diagnosis of the chronic disease?

## Abbreviations

CHD: Coronary heart disease
DM2: Diabetes mellitus type 2
GP: General practitioner
PHQ-9: Patients health questionnaire 9
PST: Problem solving treatment

## References

[1] World Health Organization. Depression fact sheet [Internet]. 2016. Available from: http://www.who.int/mediacentre/factsheets/fs369/en/.

[2] Moussavi S, Chatterji S, Verdes E, Tandon A, Patel V, Ustun B (2007). Depression, chronic diseases, and decrements in health: results from the world health surveys. Lancet.

[3] Vos T, Haby MM, Barendregt JJ, Kruijshaar M, Corry J, Andrews G (2004). The burden of major depression avoidable by longer-term treatment strategies. Arch Gen Psychiatry.

[4] van Zoonen K, Buntrock C, Ebert DD, Smit F, Reynolds CF, Beekman ATF (2014). Preventing the onset of major depressive disorder: a meta-analytic review of psychological interventions. Int J Epidemiol.

[5] Cuijpers P, van Straten A, Smit F, Mihalopoulos C, Beekman A (2008). Preventing the onset of depressive disorders: a meta-analytic review of psychological interventions. Am J Psychiatry.

[6] Muñoz RF, Cuijpers P, Smit F, Barrera AZ, Leykin Y. Prevention of major depression. Annu Rev Clin Psychol. 2010;6:181–212. 10.1146/annurev-clinpsy-033109-132040 20192789

[7] Bower P, Gilbody S. Stepped care in psychological therapies: access, effectiveness and efficiency. Narrative literature review. Br J Psychiatry. 2005;186:11–7. 10.1192/bjp.186.1.11 15630118

[8] van Straten A, Hill J, Richards DA, Cuijpers P (2015). Stepped care treatment delivery for depression: a systematic review and meta-analysis. Psychol Med.

[9] van’t Veer-Tazelaar PJ, van Marwijk HWJ, van Oppen P, van Hout HPJ, van der Horst HE, Cuijpers P (2009). Stepped-care prevention of anxiety and depression in late life: a randomized controlled trial. Arch Gen Psychiatry.

[10] Dozeman E, van Marwijk HWJ, van Schaik DJF, Smit F, Stek ML, van der Horst HE, et al. Contradictory effects for prevention of depression and anxiety in residents in homes for the elderly: a pragmatic randomized controlled trial. Int. Psychogeriatrics. 2012;24(08):1242–51. 10.1017/S1041610212000178 22436082

[11] van der Aa HP, van Rens GH, Comijs HC, Margrain TH, Gallindo-Garre F, Twisk JW, et al. Stepped care for depression and anxiety in visually impaired older adults: multicentre randomised controlled trial. BMJ. 2015;351:h6127. doi:10.1136/bmj.h6127. 10.1136/bmj.h6127 PMC4655616 26597263

[12] Zhang DX, Lewis G, Araya R, Tang WK, Mak WWS, Cheung FMC (2014). Prevention of anxiety and depression in Chinese: a randomized clinical trial testing the effectiveness of a stepped care program in primary care. J Affect Disord.

[13] Van der Weele GM, De Waal MWM, Van den Hout WB, De Craen AJM, Spinhoven P, Stijnen T (2012). Effects of a stepped-care intervention programme among older subjects who screened positive for depressive symptoms in general practice: the PROMODE randomised controlled trial. Age Ageing.

[14] van Beljouw IM, van Exel E, van de Ven PM, Joling KJ, Dhondt TD, Stek ML, et al. Does an outreaching stepped care program reduce depressive symptoms in community-dwelling older adults? a randomized implementation trial. Am J Geriatr Psychiatry. 2014;23:807–17. 10.1016/j.jagp.2014.09.012 25499673

[15] Bot M, Pouwer F, Ormel J, Slaets JPJ, de Jonge P. Predictors of incident major depression in diabetic outpatients with subthreshold depression. Diabet Med. 2010;27:1295–301. 10.1111/j.1464-5491.2010.03119.x 20950389

[16] Craig P, Dieppe P, Macintyre S, Michie S, Nazareth I, Petticrew M (2008). Developing and evaluating complex interventions: the new medical research council guidance. Br Med J.

[17] van Dijk SEM, Pols AD, Adriaanse MC, Bosmans JE, Elders PJM, van Marwijk HWJ, et al. Cost-effectiveness of a stepped-care intervention to prevent major depression in patients with type 2 diabetes mellitus and/or coronary heart disease and subthreshold depression: design of a cluster-randomized controlled trial. BMC Psychiatry. 2013;13:128. 10.1186/1471-244X-13-128 PMC3654943 23651614

[18] Kroenke KSR (2002). The PHQ-9: a new depression diagnostic and severity measure. Psychiatr Ann.

[19] Lamers F, Jonkers CCM, Bosma H, Penninx BWJH, Knottnerus JA, van Eijk JTM. Summed score of the patient health questionnaire-9 was a reliable and valid method for depression screening in chronically ill elderly patients. J Clin Epidemiol. 2008;61:679–87. 10.1016/j.jclinepi.2007.07.018 18538262

[20] Sheehan DV, Lecrubier YSK (1998). The mini-international neuropsychiatric interview (MINI): the development and validation of a structured diagnostic psychiatric interview for DSM-IV and ICD-10. J Clin Psychiatr.

[21] Van Vliet I, De Beurs E. Het Mini Internationaal Neuropsychiatrisch Interview (MINI). Een kort gestructureerd diagnostisch psychiatrisch interview voor DSM-IV en ICD-10-stoornissen [The Mini International Neuropsychiatric Interview (MINI). A short structured diagnostic psychiatric interview for DSM-IV and ICD-10 disorders]. Tijdschrift voor Psychiatrie. 2007;49(6):393–7. 17614093

[22] Meadows LM, Morse JM. Constructing evidence within the qualitative project. In Morse JM, Swanson JM, Kuzel AJ, editors. The nature of qualitative evidence. Thousend Oaks: Sage. 2001:187–200.

[23] Glasgow RE, Vogt TM, Boles SM (1999). Evaluating the public health impact of health promotion interventions: the RE-AIM framework. Am J Public Health.

[24] Knowles SE, Chew-Graham C, Adeyemi I, Coupe N, Coventry PA. Managing depression in people with multimorbidity: a qualitative evaluation of an integrated collaborative care model. BMC Fam Pract. 2015;16:32. 10.1186/s12875-015-0246-5 PMC4355419 25886864

[25] Dowrick C, Leydon GM, McBride A, Howe A, Burgess H, Clarke P, et al. Patients’ and doctors’ views on depression severity questionnaires incentivised in UK quality and outcomes framework: qualitative study. BMJ. 2009;338:b663. 10.1136/bmj.b663 19299474

[26] Lowe B, Unutzer J, Callahan CM, Perkins AJ, Kroenke K (2004). Monitoring depression treatment outcomes with the patient health questionnaire-9. Med Care.

[27] Murphy R, Ekers D, Webster L (2014). An update to depression case management by practice nurses in primary care: a service evaluation. J Psychiatr Ment Health Nurs.

[28] Peters S, Wearden A, Morriss R, Dowrick CF, Lovell K, Brooks J (2011). Challenges of nurse delivery of psychological interventions for long-term conditions in primary care: a qualitative exploration of the case of chronic fatigue syndrome/myalgic encephalitis. Implement Sci.

[29] Barley EA, Walters P, Tylee A, Murray J. General practitioners’ and practice nurses’ views and experience of managing depression in coronary heart disease: a qualitative interview study. BMC Fam Pract. 2012;13:1–10. 10.1186/1471-2296-13-1 PMC3276431 22221509

[30] ​Freud S. Verdere adviezen over de psychoanalytische techniek I: Over het inleiden van de behandeling. Werken 6. 1913c;184:186–205.

[31] Van Weel-Baumgarten E, Grundmeijer H, LichtStrunk E, van Marwijk H, van Rijswijk H, Tjaden B, et al. The NHG guideline Depression (second revision of the NHG guideline Depressive disorder). Huisarts & Wetenschap. 2012;55:25–9.

